# Docosahexanoic Acid Plus Vitamin D Treatment Improves Features of NAFLD in Children with Serum Vitamin D Deficiency: Results from a Single Centre Trial

**DOI:** 10.1371/journal.pone.0168216

**Published:** 2016-12-15

**Authors:** Claudia Della Corte, Guido Carpino, Rita De Vito, Cristiano De Stefanis, Anna Alisi, Stefano Cianfarani, Diletta Overi, Antonella Mosca, Laura Stronati, Salvatore Cucchiara, Massimiliano Raponi, Eugenio Gaudio, Christopher D. Byrne, Valerio Nobili

**Affiliations:** 1 Hepato-Metabolic Department, “Bambino Gesù” Children’s Hospital, IRCCS–Rome, Italy; 2 Department of Movement, Human and Health Sciences, Division of Health Sciences, University of Rome "Foro Italico"- Rome, Italy; 3 Histopathology Unit, “Bambino Gesù” Children’s Hospital, IRCCS- Rome, Italy; 4 Liver Research Unit, “Bambino Gesù” Children’s Hospital, IRCCS–Rome, Italy; 5 Endocrinology and Diabetes Unit, "Bambino Gesù" Children's Hospital, IRCCS—Rome, Italy; 6 Department of Anatomical, Histological, Forensic Medicine and Orthopedics Sciences, Sapienza University of Rome—Rome, Italy; 7 Department of Cellular Biotechnology and Hematology, Sapienza University Hospital Umberto I, Rome, Italy; 8 Department of Pediatrics, Pediatric Gastroenterology and Liver Unit, Sapienza University Hospital Umberto I, Rome, Italy; 9 Medical Directorate, "Bambino Gesù" Children's Hospital, IRCCS—Rome, Italy; 10 Human Development and Health Academic Unit, Faculty of Medicine, University of Southampton-, Southampton, United Kingdom; 11 NIHR Southampton Biomedical Research Centre, University Hospital Southampton NHS Foundation Trust and University of Southampton, Southampton, United Kingdom; Kaohsiung Medical University, TAIWAN

## Abstract

**Background:**

There are no licensed treatments for non alcoholic fatty liver disease (NAFLD) in adults or children. In NAFLD, several studies have shown a benefit of omega-3 fatty acid treatment on lipid profile, insulin-sensitivity and hepatic steatosis and it has also been suggested that Vitamin D treatment has potential antifibrotic properties in liver disease.

**Trial Design:**

To date, however, there are no studies that have tested the combination of Docosahexanoic acid (DHA) and vitamin D treatment which may benefit the whole spectrum of disease in NAFLD. Our aim therefore, was to test the effect of daily DHA (500 mg) plus vitamin D (800 IU) treatment, in obese children with biopsy-proven NAFLD and vitamin D deficiency, in a randomized, double-blind placebo-controlled trial.

**Methods:**

The 41/43 patients completed the study (18-treatment, 23-placebo). At 12 months: i) the main outcome was liver histology improvement, defined by NAS; ii) the secondary outcome was amelioration of metabolic parameters.

**Results:**

DHA plus vitamin D treatment reduced the NAFLD Activity Score (NAS), in the treatment group (5.4 v1.92; p<0.001 for baseline versus end of study). There was no change in fibrosis score, but a reduction of the activation of hepatic stellate cells (HSC) and fibrillar collagen content was noted (3.51±1.66 v. 1.59±1.37; p = 0.003) in treatment group. Moreover, the triglycerides (174.5 vs. 102.15 mg/dl), ALT (40.25 vs. 24.5 UI/l) and HOMA-IR (4.59 vs. 3.42) were all decreased with treatment.

**Conclusion:**

DHA plus vitamin D treatment improved insulin-resistance, lipid profile, ALT and NAS. There was also decreased HSC activation and collagen content with treatment.

## Introduction

Following the epidemic of obesity and metabolic syndrome recorded in children and adolescents in the last couple of decades, nonalcoholic fatty liver disease (NAFLD) has become the main cause of chronic liver disease in these groups. In Western countries, the prevalence of NAFLD is 20–30% in the pediatric population and 70–80% in obese children [1]. NAFLD is considered a “multi-hit” disorder, in which genetic, epigenetic and environmental factors interact causing the onset and progression of liver damage [2]. Recent studies have demonstrated that approximately 25% of children with NAFLD have NASH and interesting data derived from longitudinal studies have indicated that hepatic fibrosis is the most important prognostic marker of progression of liver disease [3,4]. The rate of progression of liver disease in NAFLD is slow with an estimated average of 7 years elapsing between the development of NASH with fibrosis in patients who had prior nonalcoholic fatty liver [5,6]. Because the presence of liver fibrosis predicts liver-related outcomes and mortality, blocking mechanisms of fibrogenesis is a key therapeutic goal in the treatment for NASH [6]. Fibrosis is characterized by an excessive deposition of extracellular matrix (ECM), with increases in total collagen content and in fibril-forming collagens (Type I, III and IV) [7]. These changes induce dysfunction and activation of the hepatic stellate cells (HSCs) with development and progression of fibrogenesis.

Lifestyle interventions, consisting of a weight decreasing diet and increases in physical exercise, remain the cornerstone of treatment of pediatric NAFLD, even if several studies indicate improvement only in metabolic parameters and liver steatosis [8,9]. Consequently, in the last decade, several pharmacological approaches have been tested that are focused on ameliorating mechanisms of liver damage. Unfortunately, none of the tested drugs to date has produced unequivocal results with the most effective treatments showing limited efficacy and worrying side effects in studies in adults. Omega-3 fatty acid treatment is potentially safe in adults and children, and docosahexaenoic acid (DHA) treatment in children and omega-3 fatty acid treatment producing > 2% DHA tissue enrichment in adults has shown promising results to decrease liver fat in patients with NAFLD [10–12].

Recently, vitamin D deficiency (VDD) has been associated with obesity, metabolic syndrome and cardiovascular risk in adults and children [13]. VDD occurs frequently among healthy children, with a rate of 55% in the American pediatric population [14]. Moreover, several studies have reported that VDD is common in patients with NAFLD and importantly, VDD is associated with increased risk of steatosis, necroinflammation and fibrosis in both adults and children with biopsy-proven NAFLD [15–18]. Several studies in humans and in animal models indicate that VDD contributes to increased oxidative stress and systemic inflammation [19]. Furthermore, emerging evidence suggests a role for VDD in fibrogenesis, with the potential therefore for an anti-fibrotic effect of vitamin D treatment [20]. The available data to date suggests that vitamin D may reduce fibrotic processes inhibiting the expression of transforming growth factor beta (TGF-β) and suppressing the deposition of collagen Iα1 and the activation of alpha-smooth muscle actine (α-SMA) positive HSCs [21].

Therefore, given the potential benefits of both DHA treatment and vitamin D treatment to ameliorate the features of NAFLD/NASH, the aim of the present study was to undertake a proof of concept, randomized double blind placebo-controlled trial (RCT) to test the potential efficacy and tolerability of a mixture of DHA and Vitamin D in children and adolescents with vitamin D deficiency and biopsy-proven NAFLD

## Materials and Methods

### Study population and design

An RCT was undertaken to examine the efficacy and safety of a mixture of vitamin D (800 IU) and DHA (500 mg) orally once daily, versus identical placebo for 24 weeks on hepatic histology and metabolic parameters in children and adolescents with biopsy-proven NAFLD. Sixty-six white European patients (4–16 years) with liver biopsy-proven NAFLD, referred to the Hepato-Metabolic Department of “Bambino Gesù” Children’s Hospital (Rome, Italy) between March 2014 and April 2015, were evaluated for the present study. Patients were recruited and studied between March 2014 and April 2015.

Children were eligible for the study if they were between: 4 and 16 years of age, had a liver biopsy result consistent with a diagnosis of NAFLD/NASH, and also had decreased serum vitamin D levels (< 20 ng/ml), aminotransferases (ALT) levels <10 upper limit of normal (ULN), and no laboratory and/or clinical signs of liver decompensation. Moreover, in all children other causes of liver disease, such as viral liver disease, autoimmune hepatitis, Wilson's disease, α-1-antitrypsin deficiency, celiac disease, alcohol consumption (any quantity), use of drugs known to induce fatty liver, were also excluded.

Patients were randomized to receive capsules combining 500 mg of docosahexaenoic acid and 800 IU of Vitamin D (Treatment arm) or identical capsules as placebo (Placebo arm).

The dosage of the DHA and vitamin D intervention were determined based on the available evidence in obese patients with NAFLD. As previously reported by our group, DHA supplementation improves liver steatosis and insulin sensitivity in children with NAFLD with similar effects for doses of 250 and 500 mg/day [22]. As for vitamin D, several expert groups, including the American Academy of Pediatrics, have recently revised the recommended supplementation dosages. In this position paper, 600–1.000 UI/day of vitamin D have been recommended in adolescents with risk factors for vitamin D deficiency, such as obese adolescents group [23]. Based on these finding, we treated our patients with 800 UI/day in the treatment arm.

A computer-generated randomization sequence assigned participants in a 1:1 ratio to treatment with Vitamin D plus DHA (Treatment arm) or placebo (Placebo arm). A statistician, who was blinded to participants' clinical data and did not participate in patients' clinical care, generated the allocation sequence and assigned participants to their group. Only the statistician had access to the treatment codes. The capsules were taken every day for 24 weeks. Additionally, all patients were included in a lifestyle intervention program consisting of a hypocaloric diet (25–30 Kcal/kg/day) and regular physical exercise (twice weekly 1-hour physical activity). Participants and investigators were blinded to the treatment for the duration of the study. Capsules were dispensed at the baseline visit, and after three months. The compliance with treatment was monitored at each visit by counting the returned capsules. Moreover, at each visit, adverse effects were recorded by the Principal Investigator. Anthropometric measurements and laboratory data were collected at each visit (at baseline, 6 and 12 months). Liver biopsy was performed at baseline and after 12 months, only in the treatment arm. For ethical reasons and according to the Position Paper of the Hepatology Committee of ESPGHAN (European Society of Pediatric Gastroenterology, Hepatology and Nutrition) at the end of study, it was decided to repeat liver biopsy only in treated patients [24] and patients in the placebo group did not undergo an end of study biopsy.

We defined changes in NAS as the primary outcome of the present proof of concept trial because several studies have showed liver histology as the most appropriate endpoint to define efficacy in clinical trials in NAFLD [25]. The secondary outcomes were the improvement of metabolic parameters, such as gluco-insulinemic profile and serum lipid concentrations.

### Anthropometrical and biochemical measurements

Anthropometric measurements and laboratory tests, including liver enzymes, gluco-insulinemic profile and lipids were performed at baseline and repeated at 6 and 12 months.

The body weight and height were measured with the patients wearing underwear. Body mass index (BMI = kg/m^2^) and standard deviation score (Z score) were calculated [26].

Serum glucose, lipid profile [triglycerides, cholesterol-total, high-density lipoprotein (HDL) cholesterol and low-density lipoprotein (LDL)], liver function tests (aspartate- (AST) and alanine- (ALT) aminotransferases, gamma-glutamyl-transpeptidase (GGT), albumin and International Normalized Ratio (INR)), fasting plasma glucose and insulin were measured in all patients after an overnight 12-h fasting. In all patients, Oral Glucose Tolerance Tests (OGTT) were performed [27]. Insulin-resistance (IR) was assessed by the homeostatic model assessment (HOMA) [HOMA-IR = (insulin0 (μIU/ml) x glucose0 (mmol/l))/22.5)]. A cut-off value of > 2.5 was considered as an index of insulin resistance [28].

In all patients, serum 25-hydroxyvitamin D [25(OH)D, vitamin D] concentration was measured by radioimmunoassay (IDS Immunodiagnostics, IDS Limited, Tyne and Wear, UK). Subjects were categorized as having either low vitamin D levels (<20 ng/mL), or normal vitamin D levels (≥20 ng/mL) [29].

### Determination of total monthly hours of sunlight

The mean hours of sunshine was determined using the “Italian atlas of solar radiation” from the ENEA center (http://www.solaritaly.enea.it). The formula for estimating mean hours of sunlight was: % Sunshine x [(Clear days x 0.85) + (Partly Cloudy days x 0.45) + (Cloudy day x 0.10) x 24]

Sunshine % = the percentage of the daylight hours for Rome during that month;
Clear days = defined as 70%-100% of sunshine; was used for the mean value of85% or 0.85 in the formula;
Cloudy days = defined as 30%-60% of sunshine; was used for the mean value of 45% or 0.45 in the formula;
Cloudy Days = defined as 0–20% of sunshine; was used for the mean value of 10% or 0.10 in the formula [30].

### Liver biopsy

Echo-guided liver biopsy was performed using an automatic core biopsy device (Biopince, Amedic, Sweden) with an 18-G needle, under general anesthesia [31]. A single experienced pathologist evaluated liver specimens. The histological features of steatosis (0–3), lobular inflammation (0–3), and hepatocyte ballooning (0–2) were combined in the NAFLD activity score (NAS), ranging from 0 to 8 using the criteria of NAFLD Clinical Research Network [32].

### Assessment of fibrillar collagen deposition in liver biopsies

The assessment of fibrillar collagen deposition within the liver biopsy was evaluated in Sirius Red (SR) stains, as previously [33,34]. Briefly, SR stained slides were scanned by a digital scanner (Aperio Scanscope CS System, Aperio Technologies, Inc, Oxford, UK) and processed by ImageScope. An image analysis algorithm has been used to quantify the proportion of SR-stained area. The algorithm was applied on the entire section (Part A and B of **S1 Fig**). The extent of collagen deposition was expressed as the proportion (%) of SR-stained area with respect to the total biopsy area, providing a quantitative value on a continuous scale. Only biopsies containing at least 5 portal tracts were considered.

In order to establish reference values for fibrillar collagen in normal liver samples, specimens from 6 lean, non-diabetic children (boys, 4; girls, 2; median age: 13 years, range, 12–16 years) without liver disease were used as controls, as previously [35]. These fragments were obtained from patients who underwent laparotomy or laparoscopic procedures (for cholecystectomy), from liver donors (orthotopic liver transplantation) or incidental “normal” liver biopsies (children exhibiting persistent/intermittent elevations of liver enzymes for >6 months). Informed consent in writing was obtained from next of kin, caretakers, or guardians on behalf of the children enrolled in this study [35,36].

### Immunohistochemistry for α smooth muscle actin and evaluation of hepatic stellate cell/myofibroblast pool

Sections were incubated overnight at 4°C with primary antibodies against α smooth muscle actin (αSMA: Dako, mouse monoclonal, code: M0851, dilution: 1:50). Samples were then incubated for 20 minutes at room temperature with secondary biotinylated antibody and, successively, with streptavidin-Horse radish peroxidase (LSAB+, Dako, code K0690). Diaminobenzidine (Dako, code K3468) was used as the substrate and the sections were counterstained with hematoxylin. For all immunoreactions, negative controls (the primary antibody was replaced with pre-immune serum) were also included.

Sections were examined with a Leica Microsystems DM 4500 B Microscopy (Weltzlar, Germany) equipped with a Jenoptik Prog Res C10 Plus Videocam (Jena, Germany). Observations were processed with an Image Analysis System (IAS, Delta Sistemi, Rome, Italy) and were independently performed by 2 researches in a blinded fashion. Only biopsies containing at least 5 portal tracts were considered.

The activation of Hepatic Stellate Cell (HSC)/Myofibroblast (MF) pool was evaluated by counting the number of αSMA-positive cells per high power field (HPF: at 40x). Perisinusoidal HSCs and portal/septal MFs were separately evaluated [36,37]; αSMA-positive HSCs were recognized in accordance with their stellate/spindle shape and their perisinusoidal location within the parenchymal lobule; besides, portal/septal MFs were considered as stellate- or spindle-shaped (αSMA-positive cells) located at the interface between parenchyma and portal tract or between parenchyma and septa, and those residing in the portal tracts and the fibrotic septa. The number of αSMA-positive HSCs and MFs was counted and expressed as number of positive cells per HPF. Only the cells which displayed nuclei on the section were considered. For each slide, at least 15 non-overlapping microscopic HPFs were randomly chosen.

### Ethical Approval

The trial was fully approved by the Ethics Committee of the Bambino Gesù Children's Hospital in January 2014; protocol number: 791.13/0PBG, see S1 Protocol), according to the Declaration of Helsinki (as revised in Seoul, Korea, October 2008) and CONSORT guidelines (see S1 Checklist). A written informed consent to the study protocol and to publication of results was obtained from the parents or legal guardians of the children. This study was registered on March 24, 2014 in ClinicalTrials.gov (Registration Number: NCT02098317 –see S1 Clinical Trial).

The authors confirm that all ongoing and related trials for this drug/intervention are registered.

### Statistical analysis

The data were analyzed using a STATISTICA (version 2010, Chicago, IL, USA). Continuous variables were expressed as mean ± standard deviation (SD). Data distribution was checked for normality by the Kolmogorov-Smirnov test. Data were analyzed using the intention-to-treat principle and the values recorded at baseline were compared to values recorded at 6 and 12 months in all patients, regardless of treatment duration. Baseline and follow up characteristics were tested for differences by Student’s *t*-test (p<0.05). The change of anthropometrical and laboratory values, between placebo and treatment groups, was evaluated using analysis of variance (ANOVA) with repeated measures. Difference between proportions were tested using the Chi-square test. Univariate correlations were investigated with Pearson’s correlation. Multivariable logistic regression analysis was used to test the independence of associations between end of study vitamin D concentrations as the key exposure and histological characteristics after adjusting for BMI, change in BMI between baseline and follow up and basal values of Vitamin D.

## Results

### Baseline characteristics

**Fig 1:**
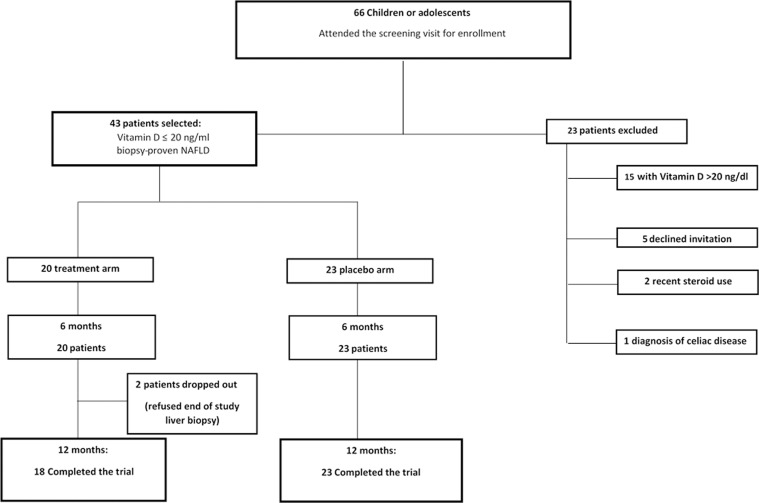
The enrollment flow-chart.

In our study, between March 2014 and April 2015, 66 patients were screened and 43 of these with biopsy-proven NAFLD were enrolled (**Fig 1**). The patients were enrolled with similar proportions recruited during the winter (20/43, 47%) and spring (23/43, 53%) months.

Twenty patients received an oral dose of 500 mg of DHA and 800 IU/day of Vitamin D (treatment arm) and 23 children received the capsules of placebo for 6 months (placebo arm). Forty-one patients completed the study, with two patients from the treatment arm being lost to follow up. There were no significant adverse events. The dropouts from the treatment arm were not associated with any study adverse events, but were due to the refusal by parents to consent to the second liver biopsy at 12 months. The two groups had similar baseline characteristics, as shown in **Table 1**.

**Table 1:** Clinical and laboratory variables in placebo and treatment arms at baseline and 12 months.

	**Arm**	**Baseline**	**6 months**	**12 months**	**Difference between groups**	**p placebo**	**p treatment**
**Sex (M/F)**	*Placebo*	10–13	10–13	10–13			
	*Treatment*	9–11	9–11	8–10			
**Age (years)**	*Placebo*	13.20 (2.16)	14.8 (3.26)	14.3 (2.16)	F_(5,18)_ = 0.45; p = 0.79	0.11	
	*Treatment*	12.30 (2.07)	12.75 (2.42)	13.05 (1.77)			0.16
**BMI, Kg/mq**	*Placebo*	28.39 (5.42)	28.22 (5.33)	28.02 (5.63)	F_(5,18)_ = 6.55; p = **0.01**°	0.33	
	*Treatment*	28.42 (4.08)	27.01 (4.77)	24.58 (3.61)			*0*.002*
**z-BMI**	*Placebo*	2.34 (0.89)	2.10 (0.75)	2.07 (0.67)	F_(5,19)_ = 0.24; p = **0.04**°	0.26	
	*Treatment*	2.16 (0.64)	1.96 (0.35)	1.67 (0.56)			*0*.05*
**WC, cm**	*Placebo*	89.95 (9.91)	89.45(8.77)	89.15 (9.74)	F_(5,19)_ = 1; p = 0.07	0.85	
	*Treatment*	89.47 (9.35)	86.91 (11.61)	85 (7.29)			0.76
**AST, UI/L**	*Placebo*	33.05 (18.72)	36.7 (28.87)	35.05 (36.98)	F_(5,18)_ = -1.45; p = **0.05**°	0.47	
	*Treatment*	28.55 (10.51)	27.64 (15.54)	20 (23.76)			0.21
**ALT, UI/L**	*Placebo*	51.20 (52.97)	45.11 (11.12)	43.45 (17.10)	F _(5,18)_ = 4.34; p = **0.0003**°	0.76	
	*Treatment*	40.25 (24.59)	34.29 (33.08)	24.5 (16.58)			*0*.013*
**GGT, UI/L**	*Placebo*	21.88 (13.45)	20.11(13.4)	18.78 (14.33)	F_(5,18)_ = 1.8; p = 0.44	0.52	
	*Treatment*	20.05 (12.92)	21.29 (12.91)	18.5 (18.12)			0.22
**Total Cholesterol, mg/dl**	*Placebo*	154.45 (30.85)	153.1 (15.44)	143.35 (18.41)	F_(5,19)_ = 0.68; p = 0.64	0.59	
	*Treatment*	163 (27.28)	155.64 (23.83)	157 (25.77)			0.23
**LDL Cholesterol, mg/dl**	*Placebo*	95.36 (32.74)	100.12 (25.6)	95.38 (33.7)	F_(5,19)_ = 3.07; p = **0.013**°	0.88	
	*Treatment*	112.05 (24.28)	107.29 (23.07)	105.5 (22.24)			0.08
**HDL Cholesterol, mg/dl**	*Placebo*	46.55 (8.53)	42.55 (6.24)	47.51(8.55)	F_(5,19)_ = 4; p = **0.07**°	0.87	
	*Treatment*	34.5 (8.55)	41.88 (6.82)	43.77 (7.31)			*0*.008*
**Triglycerides, mg/dl**	*Placebo*	87.20 (47.40)	88.94 (41.33)	89.44 (44)	F_(5,19)_ = 10.1; p<**0.00001**°	0.43	
	*Treatment*	174.5 (75.63)	127.35 (64.30)	102.15 (22.24)			*<0*.0001*
**Glucose, mg/dl**	*Placebo*	82.50 (7.36)	85.7 (4.18)	80.80 (6.27)	F_(5,18)_ = 0.89; p = 0.46	0.52	
	*Treatment*	84.85 (6.44)	80.5 (13.08)	77.82 (8.91)			0.49
**Glucose-120’**	*Placebo*	100.2 (13.12)	101.2 (12.23)	97.10 (10.21)	F_(5,18)_ = 0.61; p = 0.69	0.77	
	*Treatment*	102.54 (13.31)	101.8 (11.23)	103 (26.69)			0.94
**Insulin, mU/L**	*Placebo*	22.31 (14.74)	23.44 (16.4)	21.71 (12.23)	F_(5,19)_ = 0.31; p = 0.79	0.96	
	*Treatment*	25.03 (21.22)	23.13 (13.60)	21.16 (15.46)			0.33
**Insulin -120’**	*Placebo*	77.16 (40.99)	86.3 (24.35)	84.63 (31.53)	F_(5,19)_ = 2.97; p = **0.015**°	0.74	
	*Treatment*	123.21 (83.72)	126.99 (70.46)	92 .93 (50.1)			*0*.04*
**HOMA-IR**	*Placebo*	4.56 (3.13)	4.28 (2.64)	4.33 (2.52)	F_(5,19)_ = 1.27; p = 0.29	0.73	
	*Treatment*	4.59 (4.26)	4.29 (2.69)	3.42 (2.90)			*0*.05*
**HbA1c, mmol/mol**	*Placebo*	35.31(1.21)	35.22 (1.99)	36.42 (2.96)	F_(5,19)_ = 1.27;p = 0.73	0.78	
	*Treatment*	34.54 (1.34)	35.48 (1.61)	36.09 (1.45)			0.12
**Uric Acid, mg/dl**	*Placebo*	5.44 (1.67)	6.01 (1.11)	5.62 (2.96)	F_(5,18)_ = 2.46;p = 0.09	0.29	
	*Treatment*	5.86 (1.25)	6.27 (2.14)	6.10 (1.15)			0.16
**Vitamin D, ng/ml**	*Placebo*	16.98 (3.47)	17.01 (3.17)	18.36 (3.87)	F_(5,18)_ = 16; p = <**0.0001**°	0.86	
	*Treatment*	15.98 (5.03)	29.7 (6.21)	25.42 (4.72)			*0*.001*

BMI = Body mass index, z-BMI: z-score body mass index, WC = waist circumference, ALT = Alanine aminotransferase, AST = aspartate aminotransferase, GGT = γ-glutamyltransferase,HDL = high-density lipoprotein cholesterol, LDL = low-density lipoprotein cholesterol, HOMA-IR = homeostatic model assessment of insulin resistance, HbA1c glycosylated haemoglobin. Data are expressed as means ± standard deviation (SD). Standard deviation: average standard deviation of blood glucose of the patients during the 24-hour monitoring period.

* ANOVA (p<0.05) between baseline and 12 months.

° ANOVA with repeated measures.

### Effects on anthropometric, clinical and laboratory parameters

**Table 1**shows the anthropometric and laboratory characteristics for each arm of the study. At 12 months, the placebo group showed no significant improvements for any anthropometric and laboratory parameters. In the treatment arm, at 12 months, there was a decrease in BMI (28.42 to 24.58 kg/m^2^, p = 0.04), serum triglyceride concentration (174.5 to 102.15 mg/dl; p = 0.001) and in the measure of insulin-resistance were observed (HOMA-IR 4.59 to 3.42; p = 0.03). Repeated measures ANOVA showed both treatment and placebo decreased BMI (F_(5,18)_ = 6.55; p = 0.0001), ALT (F_(5,18)_ = 4.34; p = 0.0003), Triglycerides (F_(5,19)_ = 10.1; p = 0.0001), insulin-‘120 (F_(5,19)_ = 2.97; p = 0.015) and vitamin D (F_(5,18)_ = 16; p<0.0001).

### Vitamin D supplementation

At baseline, all patients showed vitamin D deficiency (VDD), with median values of vitamin D of 16.01±3.98 ng/dL. The values of vitamin D were normalized to the hours of sunlight. In the placebo arm, the values of vitamin D did not change during the study, with persistent VDD. In contrast, in the treatment group, a persistent and significant increase of Vitamin D concentration was observed (baseline = 15.98; 6-months = 29.7 and 12-months = 25.42 ng/dL; p = 0.02). None of treated patients developed hypercalcemia and/or nephrotoxicity.

### Effects on liver histology

Improvement in liver histology was the primary outcome of the trial. **Table 2**showed all histological features and NAS scores assessed at baseline in both groups and after 12 months in the treatment group. The data were similar between the two groups at baseline for steatosis, ballooning, portal and lobular inflammation and fibrosis. Lower levels of 25 (OH) D3 were associated with greater fibrosis and steatosis. Before randomization, the biopsies were classified, in accordance with the NASH CRN-criteria, into Not-NASH (N = 6) and definite NASH (N = 14). After treatment with DHA and Vitamin D, the classification of biopsies indicated a decrease of definite NASH (N = 3) and an increase of not-NASH diagnosis (N = 17). Moreover, NAS improved (from 5.40 to 1.92; p<0.001), and steatosis (from 2.25 to 1.0; p = 0.002), ballooning (from 1.6 to 0.46; p = 0.001), lobular inflammation (from 1.5 to 0.88; p = 0.04) and portal inflammation (from 1.6 to 1.0; p = 0.05)], whilst there was a trend toward a decrease in fibrosis (from 2.0 to 1.5; p = 0.06). Fibrosis severity at baseline was: stage 1c in 13 samples, stage 2 in 6, stage 3 in 1, and there were no biopsies that were classified as fibrosis stage 4. After treatment, no statistical significant changes were present in fibrosis stage [stage 1c: 12 patients; stage 2: 6 patients; (stages 3–4: no patients)].

**Table 2:** Liver histology characteristics in treatment and placebo arm at baseline for both groups and end of study (12 months)2 in the treatment arm.

	Baseline			12 months	
	Placebo	Treatment		Treatment	
Number (%)	23 (100)	20 (100)	p^a^	18 (90%)	p^b^
Steatosis					
0	/	/	/	5 (35.70)	/
1	9(39.13)	/	/	9 (6.30)	/
2	8(34.77)	13(65)	0.06	/	/
3	6(26.1)	7(35)	0.31	/	/
Lobular Inflammation					
0	2(8.7)	/	/	3 (21.43)	/
1	13 (56.52)	15 (75)	0.62	11 (78.57)	0.62
2	8 (34.78)	5 (25)	0.44	/	/
Portal inflammation					
0	2(8.7)	/	/		
1	15 (65.2)	14 (70)	0.91	14 (100)	0.08
2	6 (26.1)	6 (30)	0.87	/	/
Ballooning					
0	1(4.35)	/	/	8 (57.15)	/
1	14(60.87)	13(65)	0.44	6 (42.85)	0.41
2	8 (34.78)	7 (35)	/	/	/
NAS					
1	/	/	/	5(35.70)	/
2	1(4.35)	/	/	5(35.70)	/
3	6(26.12)	/	/	4(28.60)	/
4	4(17.4)	6(30)	0.21	/	/
5	8 (34.78)	10 (50)	0.76	/	/
6	3(13)	3(15)	0.99	/	/
7	1 (4.35)	1 (5)	0.98	/	/
Fibrosis					
0	/	/	/	/	/
1c	18(78.3)	13(65)	0.09	12(66.6)	0.92
2	5(21.7)	6(30)	0.12	6(33.4)	0.99
3	/	1(5)	/	/	/
**α-SMA+ cells**		**(Means ± SD)**			
pericentral HSCs	/	8.64±4.92	/	2.72±2.51	**< 0.01**
portal MFs	/	3.94±1.78	/	2.05±1.49	**< 0.05**

α-SMA = α smooth muscle actin; HSCs = Hepatic Stellate Cells; MFs = Myofibroblasts; SD = Standard Deviation

a: placebo vs. treatment to baseline

b: treatment baseline vs. treatment 12 months

Binary logistic regression analysis showed that change in Vitamin D level with treatment was independently associated with features of NAFLD as dichotomous outcomes [fibrosis (OR = 2.96, 95% CI = 1.9–4.69, p-value = 0.003), steatosis (OR = 3.53, 95% CI = 1.33–3.4, p-value = 0.001) and NAS (OR = 2.75, 95% CI = 1.2–3.32, p-value = 0.005) (**Table 3**)].

**Table 3:** Binary logistic regression showing the effect of increase in vitamin D levels with treatment on change in liver histology characteristics.

Variables	Unadjusted OR (95% CI)^a^	*p*-value	Adjusted OR (95% CI)^b^	*p*-value
Ballooning	3.7(0.99–4.9)	0.28	2.1(0.78–3.6)	0.13
Fibrosis	5.48(2.01–8.7)	**0.001**	2.96 (1.9–4.69)	**0.003**
Steatosis	6.77(2.79–10.4)	**0.001**	3.53(1.33–3.4)	**0.001**
Lobular Inflammation	5.44(1–7.99)	0.25	2.5(0.93–3.3)	0.11
Portal Inflammation	4.74(2.44–5.77)	**0.05**	1.82(1.22–2.33)	**0.05**
NAS score	4.89(2.11–8.64)	**0.006**	2.75(1.2–3.32)	**0.005**

a: unadjusted analysis

b: adjusted for BMI and basal Vitamin D concentration

### Fibrosis and collagen deposition assessment in liver biopsies

Since the available evidence suggests that vitamin D may have a beneficial effect on fibrogenesis and as we observed a trend toward an improvement in fibrosis score with treatment, further exploratory analyses were undertaken to examine the effects of treatment on factors involved in the fibrogenetic process. The fibrillar collagen content was assessed in SR stained biopsies at baseline; overall, NAFLD biopsies at baseline showed increased but not statistically significant values of fibrillar collagen content (2.60 ± 1.76), compared with normal controls (1.44 ± 0.41; p = 0.088). Only eleven out of twenty NAFLD biopsies at baseline showed increased content of collagen fibers (3.51 ± 1.66) in comparison with normal samples (p<0.01). Moreover, biopsies with a fibrosis score = 2/3 (N = 7) had higher fibrillar collagen content (4.17 ± 2.10) in comparison with those obtained from patients with fibrosis score = 1 (1.90 ± 1.11; p< 0.05).

At the baseline, the fibrillar collagen content calculated in SR stained slides was significantly correlated with fibrosis stage (r = 0.647; p<0.02) and NAS score (r = 0.736; p<0.01).

**Fig 2:**
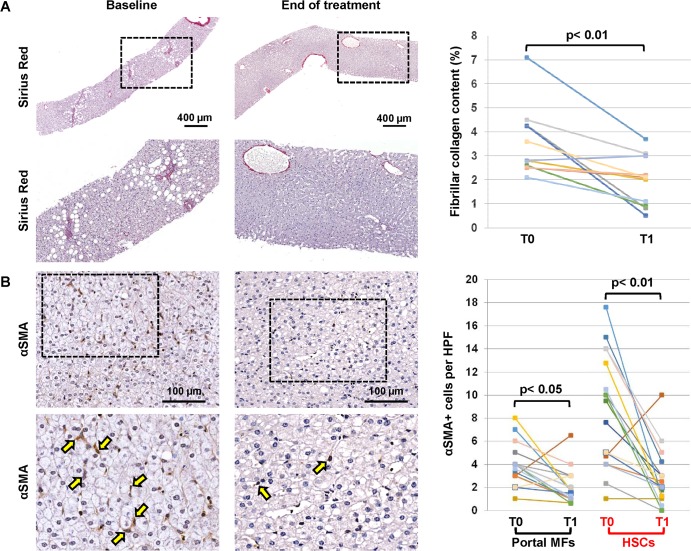
Assessment of fibrillar collagen content and activation of hepatic stellate cells (HSCs) in liver biopsies. (A) The fibrillar collagen content was assessed in Sirius Red (SR) stained biopsies. Patients with an increased collagen content at the baseline (N = 11) showed a significant decrease in fibrillar collagen content at the end of the treatment. (B) The activation of HSCs and portal Myofibroblasts (MF) was evaluated by immunohistochemistry for α Smooth Muscle Actin (αSMA). At the end of the treatment, the number of αSMA+ HSCs and portal MFs was significantly reduced in comparison with biopsies at the baseline.

Patients with an increased fibrosis content at the baseline (N = 11) showed a significant decrease in fibrillar collagen content at the end of the treatment (1.59 ± 1.37 v. x; paired t-test: t = 3.86 p = 0.003; **Fig 2**).

#### Activation of HSC/MF pool

The activation of HSC/MF pool was evaluated at the baseline and at the end of the treatment by immunohistochemistry for αSMA.

At the end of the treatment, the number of αSMA+ HSCs/MFs was significantly reduced (pericentral HSC = 2.72±2.51 and periportal MFs = 2.05±1.49) compared with biopsies at baseline (paired t-test: t = 4.60 p< 0.01 and t = 3.53, p<0.05, **Fig 2**).

## Discussion

To the best of our knowledge, this is the first RCT evaluating the efficacy of treatment with DHA plus Vitamin D in NAFLD/NASH patients with vitamin D deficiency, using changes in liver histology as the primary end-point. In accord with a previous study testing the effect of DHA treatment in pediatric NAFLD, the results of our study show that the administration of a mixture of DHA and vitamin D was associated with an improvement in insulin resistance with a concomitant reduction of serum triglyceride concentration and an improvement in ALT concentration. In **Table 4**we have compared the effect of treatment in the presented trial with that of our previous DHA trial in pediatric NAFLD [18], in order to test whether there were more marked effects in the DHA plus vitamin D intervention. These data show there were no significant differences between the trials for differences in triglyceride concentrations, HOMA-IR or ALT levels; thus, these comparative data suggest that treatment with DHA plus vitamin D is not better than DHA treatment alone in producing an improvement in these parameters (that are often abnormal in patients with NAFLD). Therefore, the data suggest that the amelioration of the metabolic profile observed in our patients in the current trial is probably related to the DHA treatment alone, rather than to the vitamin D treatment.

**Table 4:** The percentage change in anthropometric and biochemical tests with treatment for the DHA+vitamin D intervention group in the presented study compared with the percentage change in the same parameters with DHA treatment alone [22] from a previous study.

	% change with treatment		
	DHA/vitD	DHA	P
BMI, Kg/m^2^	-13.51 ±2.1	-4.13±3.1	**0.02**
WC, cms	-4.99 ±1.1	-5.17±2.4	0.67
z-BMI (SDS)	-12.54±0.1	-3.98±0.4	**0.01**
AST, IU/L	-29.94±2.1	-35.41±2.5	0.12
ALT, IU/L	-39.13±3.9	-41.56±4.3	0.24
GGT, IU/L	-1.99±1.4	+1.19±1.6	0.09
Total Chol, mg/dL	-3.60±1.5	-18.87±2.4	**0.05**
LDL Cholesterol, mg/dL	-5.85±2.4	-19.63±3.3	**0.01**
HDL cholesterol, mg/dL	+21.19±1.8	-19.64±3.7	**0.02**
Triglycerides, mg/dL	-41.46±1.7	-12.98±2.4	**0.01**
Glucose-0’, mg/dL	-3.19±2.5	-7.44±3.1	0.23
Glucose-120’, mg/dl	-10.19±5.4	-14.42±2.9	0.44
Insulin-0’, mU/L	-24.57±6.2	-32.52±7.1	0.13
Insulin -120’, mU/L	-15.47±4.3	-11.65±5.4	0.75
HOMA-IR	-22.29±1.4	-39.55±1.6	0.09

The presented data are also in accord with other studies in which supplementation of vitamin D in obese children did not affect the lipid profile and markers of insulin resistance and inflammation [38,39]. In contrast to our previous trial, in the presented study we observed a reduction of BMI in the treatment arm at the end of the trial (12 months).

The greater weight decrease in the treatment arm may be due better adherence in this group of children to the therapeutic lifestyle advice that was given to all participants. It is well accepted that weight loss can improve the early features of NAFLD, but it is important to note that the benefit of the intervention was independent of weight loss in the treatment arm of the study.

Regarding our primary outcome, NAS improved in all treated patients, with a significant reduction of steatosis, ballooning, portal and lobular inflammation. In fact, 14/20 patients with NASH at baseline improved with treatment. This improvement in NAS was similar to that observed in our previous trial testing the effects of DHA treatment alone in pediatric NASH (p<0.05, **Table 5**) [35].

**Table 5:** Differences in histological characteristics with treatment with the DHA + vitamin D intervention in the presented study and the differences in histological characteristics with DHA treatment alone for comparison from a previous study [22].

	Interventon DHA + Vitamin D			Intervention DHA alone		
	Baseline	End of study	p-value	Baseline	End of study	p-value
**Steatosis**	2.25±0.42	1±0.1	0.002	1.70±1.08	0.50±0.61	<0.001
**Balloning**	1.6±0.47	0.46±0.49	<0.001	0.85±0.67	0.25±0.44	<0.001
**Lobular Infl**	1.5±0.44	0.88±0.33	0.04	1.15±0.59	0.85±0.37	<0.05
**Portal Infl**	1.6±0.45	1±0.11	0.05	-	-	-
**Fibrosis**	2±0.26	1.5±0.5	0.06	1.60±0.60	1.45±0.76	0.48
**NAS**	5.4±0.81	1.92±0.92	<0.001	3.70±1.78	1.60±1.05	<0.01

Moreover, bearing in mind the potential for benefit of vitamin D treatment on fibrosis in NAFLD and the known prognostic implication of liver fibrosis for serious chronic liver disease-related outcomes, we evaluated the effects of treatment on changes in fibrosis score in the treatment arm. In recent years, the role of vitamin D in metabolic syndrome and cardiovascular risk has attracted considerable attention and several reports suggest a crucial role for vitamin D in NAFLD development and progression. A recent systematic review demonstrated that patients with NAFLD were 1.26-times more likely to be vitamin D deficient compared with controls [15]. Moreover, both in adults and in children, studies show that low levels of vitamin D are associated with NAFLD, independently of known metabolic risk factors [18,40].

Deficiency of vitamin D may play a role in the development of fibrosis in NAFLD. For example, Zhu et al reported that long-term vitamin D deficiency can provoke chronic liver inflammation, inducing apoptosis and activation of hepatic stellate cells (HSC) to initiate liver fibrosis [41]. There is also evidence indicating that vitamin D is able to modulate HSC activation in vitro and to reduce liver fibrosis in experimental models of liver injuries [21,33]. Despite a clear and marked improvement in the NAS, there was only a non significant trend toward an improvement in fibrosis score. In keeping with this trend toward an improvement in fibrosis score, our results indicate that vitamin D administration reduces the activation of HSC/MF pool and, in patients with increased fibrillar collagen content, we observed signs of total collagen content reduction at the end of treatment.

Results from experimental cirrhosis in rats indicate that vitamin D treatment is able to prevent liver fibrosis but does not ameliorate established cirrhosis [42]. In keeping with these data, no patients included in our trial presented with bridging fibrosis or established cirrhosis at baseline. Consequently, our results are relevant only to the early phases of fibrogenesis and the data suggest there is a benefit of reduced activation of fibrogenetic cells (HSC/MF pool) after the treatment with vitamin D.

The presented study has some limitations. The first is the lack of an end of study liver biopsy in the placebo group. For ethical reasons, bearing in mind that our patients are children, the liver biopsy was not repeated at 12 months in this group. Additionally, it was not possible to test separate effects of DHA and vitamin D in this trial by using a 2x2 factorial study design and as this was a proof of concept study that lacked an end of study biopsy in the placebo group, we did not attempt a sample size calculation. A second limitation is that none of our patients showed bridging fibrosis or cirrhosis at baseline and, thus, the observed results are limited to the early stages of fibrogenesis. Therefore, it is remains uncertain whether this treatment is effective in modifying the fibrogenetic pattern in more advanced stages of liver fibrosis (F3-F4). Another limitation could be the dosage of vitamin D used in our study (800 IU daily), which although twice the average daily requirement of vitamin D, is lower than the dosage prescribed in previous clinical trials in adults with NASH. Actually, data regarding the safety of vitamin D supplementation in pediatric NAFLD is lacking. Consequently, we considered it necessary to use this dosage of vitamin D for only six months, in order to avoid possible adverse effects. For ethical reasons, we repeated the liver biopsy in our patients after one year (and not at 6 months) after randomization.

In conclusion, the results of our proof of concept study have shown beneficial effects of DHA plus vitamin D treatment on insulin-resistance, ALT triglyceride concentration and NAS score in VDD patients with biopsy-proven NAFLD. The combination of 500 mg o.d. of DHA and 800 IU o.d. vitamin D was safe over 6 months of intervention. The supplementation of a mixture of DHA and vitamin D in VDD obese children and adolescents with NAFLD may induce a remodeling of the fibrogenetic pattern with a reduction of the activation of the HSC/MF pool and of collagen content. We suggest that further longer-term studies are now warranted in both adults and children, including a greater number of patients with more advanced stage of fibrosis, in order to confirm our preliminary results.

## Supporting Information

S1 Checklist Consort Checklist. (DOC)

S1 Clinical Trial Clinical Trial Registration. (PDF)

S1 Fig **A)Method for quantification of fibrillar collagen content in Sirius Red (SR) stained slides.** SR stained slides were scanned by a digital scanner (images on the left) and processed by ImageScope. An image analysis algorithm has been used for the deconvolution of red color (SR) and stained areas are then quantified. The algorithm was applied on the entire section. The extent of collagen deposition was expressed as the proportion (%) of SR-stained area with respect to the total biopsy area, providing a quantitative value on a continuous scale. The arrows indicated fibrillar collagen content in portal areas and asterisks showed perisinusoidal accumulation of fibrillar collagen. **B) Quantification of fibrillar collagen content in Sirius Red (SR) stained slides.** An image analysis algorithm has been used for the deconvolution of red color (SR) and stained areas are then quantified before and at the end of the treatment. Representative images are represented before and after the color deconvolution processes. Patients with an increased collagen content at the baseline showed a significant decrease in fibrillar collagen content at the end of the treatment. The arrows indicated fibrillar collagen content in portal areas and asterisks showed perisinusoidal accumulation of fibrillar collagen.
(DOCX)

S1 Protocol Protocol Ethics Committee. (DOC)
