# Supplementary material for: Docosahexanoic Acid Plus Vitamin D Treatment Improves Features of NAFLD in Children with Serum Vitamin D Deficiency: Results from a Single Centre Trial
Source: PLoS One. 2016 Dec 15;11(12):e0168216. doi: 10.1371/journal.pone.0168216 (PMC5158039; doi:10.1371/journal.pone.0168216)
Supplement: S1 Clinical Trial — (PDF) [file pone.0168216.s002.pdf]

ClinicalTrials.gov
A service of the U.S. National Institutes of Health

DHA and Vitamin D in Children With Biopsy-proven NAFLD (VitD_DHA)

This study has been completed.

Sponsor:
Bambino Gesù Hospital and Research Institute

Information provided by (Responsible Party):
Valerio Nobili, Bambino Gesù Hospital and Research Institute

ClinicalTrials.gov Identifier:
NCT02098317

First received: March 24, 2014
Last updated: January 13, 2016
Last verified: January 2016
History of Changes

Tracking Information

**First Received Date** ICMJE	March 24, 2014
**Last Updated Date**	January 13, 2016
**Start Date** ICMJE	January 2014
**Primary Completion Date**	May 2015 (final data collection date for primary outcome measure)
**Current Primary Outcome Measures** ICMJE **(submitted: March 26, 2014)**	Improvement in NAFLD Activity Score (NAS) [Time Frame: 12 months] [Designated as safety issue: No]
**Original Primary Outcome Measures** ICMJE	*Same as current*
**Change History**	Complete list of historical versions of study NCT02098317 on ClinicalTrials.gov Archive Site
**Current Secondary Outcome Measures** ICMJE **(submitted: March 26, 2014)**	• improvement of laboratory parameters of metabolic syndrome, such as lipids and gluco-insulinemic profile [Time Frame: at 6 and 12 months] [Designated as safety issue: No]<br>• safety [Time Frame: 6 months] [Designated as safety issue: Yes]<br>clinical examination, medical history and specific laboratory parameters
**Original Secondary Outcome Measures** ICMJE	*Same as current*
**Current Other Outcome Measures** ICMJE	*Not Provided*
**Original Other Outcome Measures** ICMJE	*Not Provided*

Descriptive Information

**Brief Title** ICMJE	DHA and Vitamin D in Children With Biopsy-proven NAFLD
**Official Title** ICMJE	Efficacy and Tolerability of Vitamin D and Docosahexaenoic Acid (DHA) in Children With Biopsy Proven NAFLD
**Brief Summary**	

	Non-alcoholic fatty liver disease (NAFLD) has reached epidemic proportions and is rapidly becoming the one of most common causes of chronic liver disease in children. The pathogenesis of NAFLD is generally considered the result of a series of liver injuries, commonly referred as "multi-hit" hypothesis. Several studies suggest that inflammatory pathways and oxidative stress could be responsible of disease progression.<br>The purpose of this interventional study is to evaluate the efficacy and tolerability of docosahexaenoic acid (DHA) and Vitamin D in children and adolescents with biopsy-proven nonalcoholic fatty liver disease (NAFLD).
**Detailed Description**	Sixty-six children or adolescents (4-16 years) with liver biopsy proven NAFLD will be enrolled. They will be randomized to treatment with DHA and Vitamin D (n=33) or an identical placebo (n=33) given orally for a period of 6 months. All patients will be included in a lifestyle intervention program consisting of a diet tailored on the individual requirements and physical exercise.<br>Patients will undergo a medical evaluation at 3-6 and 12 months during the 12-months study period. Liver biopsy will be performed at baseline and at 12 months. Anthropometric measurements and laboratory tests, including liver enzymes, gluco-insulinemic profile and lipids will be performed at baseline and repeated at 6-12 months
**Study Type** ICMJE	Interventional
**Study Phase**	Phase 3
**Study Design** ICMJE	Allocation: Randomized<br>Endpoint Classification: Safety/Efficacy Study<br>Intervention Model: Parallel Assignment<br>Masking: Double Blind (Subject, Caregiver, Investigator, Outcomes Assessor)<br>Primary Purpose: Treatment
**Condition** ICMJE	• NAFLD<br>• Non Alcoholic Steatohepatitis (NASH)
**Intervention** ICMJE	• Drug: DHA plus Vitamin D<br>DHA 500 mg plus Vitamin D 800 IU<br>• Drug: Placebo<br>Placebo pearls mimicking pearls with DHA and Vitamin D
**Study Arm (s)**	• Experimental: TREATED GROUP<br>this group will treated with pearls containing DHA plus Vitamin D3 (500 mg and 800 IU, respectively) given orally in association with lifestyle intervention [hypocaloric diet (25-30 Kcal/kg/day) or isocaloric (40-45 Kcal/kg/day) and physical activity] for 24 weeks<br>Intervention: Drug: DHA plus Vitamin D<br>• Placebo Comparator: PLACEBO GROUP<br>this group will treated with identical placebo pearls given orally in association with lifestyle intervention [hypocaloric diet (25-30 Kcal/kg/day) or isocaloric (40-45 Kcal/kg/day) and physical activity] for 24 weeks<br>Intervention: Drug: Placebo
**Publications** *	*Not Provided*

* **Includes publications given by the data provider as well as publications identified by ClinicalTrials.gov Identifier (NCT Number) in Medline.**

Recruitment Information

**Recruitment Status** ICMJE	Completed
**Enrollment** ICMJE	66
**Completion Date**	September 2015
**Primary Completion Date**	May 2015 (final data collection date for primary outcome measure)
**Eligibility Criteria** ICMJE	Inclusion Criteria:<br>• biopsy consistent with the diagnosis of NAFLD/NASH<br>• reduced serum levels of vitamin D aminotransferases (ALT) levels <10 upper limit of normal

	• hyperechogenicity at liver ultrasound examination suggestive of fatty liver<br>• International normalized ratio (INR) < 1,3<br>• Albumin > 3 g/dl<br>• total bilirubin < 2,5 mg/dl<br>• no previous gastrointestinal bleeding<br>• no previous portosystemic encephalopathy<br>• normal renal function<br>• no hepatitis B, hepatitis C infection<br>• normal cell blood count<br>Exclusion Criteria:<br>• alcohol consumption<br>• use of drugs known to induce steatosis or to affect body weight and carbohydrate metabolism<br>• autoimmune liver disease, metabolic liver disease, Wilson's disease, and a-1-antitrypsin-associated liver disease<br>• every clinical or psychiatric disease interfering with experimentation according to investigator's evaluation<br>• finding of active liver disease due to other causes
**Gender**	Both
**Ages**	4 Years to 16 Years (Child)
**Accepts Healthy Volunteers**	No
**Contacts** ICMJE	*Contact information is only displayed when the study is recruiting subjects*
**Listed Location Countries** ICMJE	Italy
**Removed Location Countries**	

Administrative Information

**NCT Number** ICMJE	NCT02098317
**Other Study ID Numbers** ICMJE	VD3_DHA_NAFLD
**Has Data Monitoring Committee**	*Not Provided*
**Plan to Share Data**	*Not Provided*
**IPD Description**	*Not Provided*
**Responsible Party**	Valerio Nobili, Bambino Gesù Hospital and Research Institute
**Study Sponsor** ICMJE	Bambino Gesù Hospital and Research Institute
**Collaborators** ICMJE	*Not Provided*
**Investigators** ICMJE	Principal Investigator: Valerio Nobili, Professor — Bambino Gesù Children Hospital
**Information Provided By**	Bambino Gesù Hospital and Research Institute
**Verification Date**	January 2016

ICMJE **Data element required by the International Committee of Medical Journal Editors and the World Health Organization ICTRP**
