# Supplementary figures and images for: Docosahexanoic Acid Plus Vitamin D Treatment Improves Features of NAFLD in Children with Serum Vitamin D Deficiency: Results from a Single Centre Trial
Source: PLoS One. 2016 Dec 15;11(12):e0168216. doi: 10.1371/journal.pone.0168216 (PMC5158039; doi:10.1371/journal.pone.0168216)

**S1 Fig. A**

**
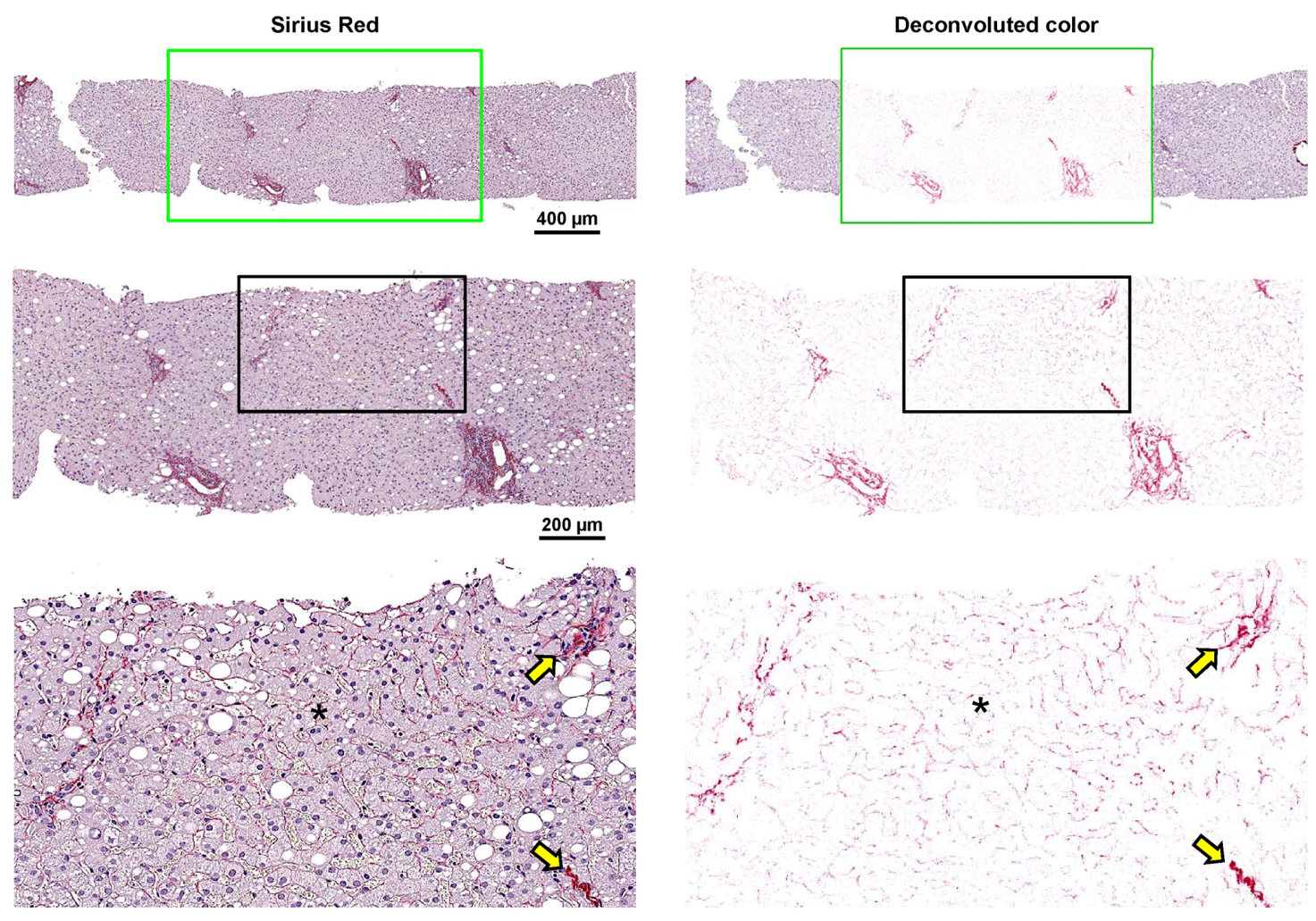
**

**S1 Fig. B**


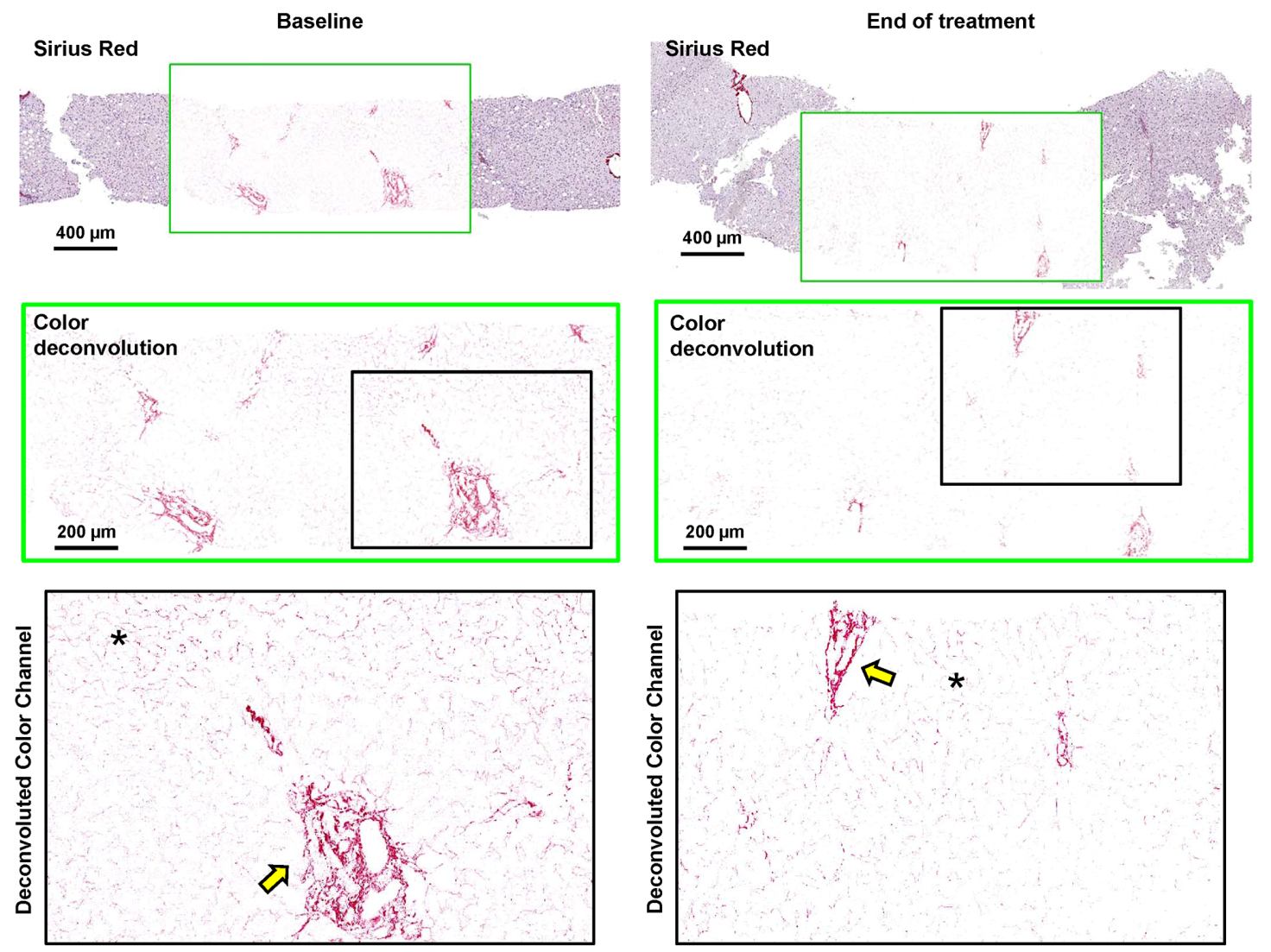

Supplement: S1 Fig — A)Method for quantification of fibrillar collagen content in Sirius Red (SR) stained slides. SR stained slides were scanned by a digital scanner (images on the left) and processed by ImageScope. An image analysis algorithm has been used for the deconvolution of red color (SR) and stained areas are then quantified. The algorithm was applied on the entire section. The extent of collagen deposition was expressed as the proportion (%) of SR-stained area with respect to the total biopsy area, providing a quantitative value on a continuous scale. The arrows indicated fibrillar collagen content in portal areas and asterisks showed perisinusoidal accumulation of fibrillar collagen. B) Quantification of fibrillar collagen content in Sirius Red (SR) stained slides. An image analysis algorithm has been used for the deconvolution of red color (SR) and stained areas are then quantified before and at the end of the treatment. Representative images are represented before and after the color deconvolution processes. Patients with an increased collagen content at the baseline showed a significant decrease in fibrillar collagen content at the end of the treatment. The arrows indicated fibrillar collagen content in portal areas and asterisks showed perisinusoidal accumulation of fibrillar collagen. (DOCX) [file pone.0168216.s003.docx]
