# Supplementary material for: Docosahexanoic Acid Plus Vitamin D Treatment Improves Features of NAFLD in Children with Serum Vitamin D Deficiency: Results from a Single Centre Trial
Source: PLoS One. 2016 Dec 15;11(12):e0168216. doi: 10.1371/journal.pone.0168216 (PMC5158039; doi:10.1371/journal.pone.0168216)
Supplement: S1 Protocol — (DOC) [file pone.0168216.s004.doc]

**Bambino Gesù Children’s Hospital, Rome**

**Unit of HepatoMetabolic Disease**

**“EFFICACY AND TOLERANCE OF TREATMENT WITH DHA AND VITAMIN D IN CHILDREN WITH NON-ALCOHOLIC STEATOHEPATITIS (NASH)”**

Title of trial:

**“EFFICACY AND TOLERANCE OF TREATMENT WITH DHA AND VITAMIN D IN CHILDREN WITH NASH”**

***Protocol Ethics Committee: 791.13/OPBG***

**PROMOTER:** a spontaneous Study

**Unit Proposing:**

HepatoMetabolic Disease Unit - Bambino Gesù Children’s Hospital, IRCCS, Rome

Dr. Valerio Nobili

**Nome of drug:** KRILLING D

**DRUG CLASS MEMBERSHIP:** Dietary food for special medical purposes

**PHARMACEUTICAL FORM:** pearls

**MEDICATION AND 'MARKETING?** YES

**IF YOU ARE INDICATING:** administration of an additional quota of omega 3 fatty acids and vitamin D to the daily diet.

**EXPERIMENTAL PHASE OF RESEARCH:** III

**INDICATIONS FOR USE OF THE DRUG:** Aim of the research will be to evaluate the efficacy and safety of treatment with DHA and vitamin D in children with non-alcoholic steatohepatitis:

- Evaluation of the tolerability of the administration of DHA and vitamin D

- Assessing the effectiveness of the mixture of DHA + Vitamin D in improving the condition score

- Evaluating the effectiveness of DHA and vitamin D in improving haematochemical 'markers' of the evolution of the metabolic syndrome

**INTRODUCTION TO TRIAL**

The non-alcoholic fatty liver disease (NAFLD) Paediatric has in recent years reached epidemic proportions. currently NAFLD affects 3-10% of normal-weight children and 70-80% of those who are overweight / obese. Over the next 10-20 years, many countries WILL doubling the frequency of the disease, making it emergency identify effective therapeutic strategies. the NASH is characterized by hepatomegaly and / or elevated aminotransferase levels and hepatic steatosis in the absence of other known causes of liver disease and / or consumption of alcohol. Histologically is characterized by inflammation and necrosis of the liver tissue. The main risk factor for the development of NASH is obesity. The pathogenesis is still not well known, several studies suggest that oxidative stress is implicated in the pathophysiology of NASH.

The recruited patients receive two capsules a day containing) docosahexaenoic acid (DHA); ii) Vitamin D3, or placebo for 24 weeks. there is provided a follow-up laboratory-instrumental at baseline (screening visit) and every 6 months up to 12 months (52 weeks).

From a clinical point of view the expected result, eliminated hepatocyte oxidative stress caused by inflammation and fibrosis, will be to interrupt the evolution of the disease to cirrhosis and organ failure.

The therapeutic options currently available for the treatment of pediatric patients with NASH are represented by diet and the increase daily physical activity.

The drugs that will be used in this study have the following characteristics:

- DHA is an essential polyunsaturated fatty acid that belongs to the very long chain Omega 3 series, of which the fish fats, derived from alpha-linoleic precursor are rich. As a precursor of eicosanoids, DHA has an anti-inflammatory enzyme effect, which modulate the metabolism of lipoproteins. For these characteristics it has been suggested activity in the prevention of metabolic syndrome.

- VITAMIN D is a fat soluble vitamin produced in the skin from 7-dehydrocholesterol upon exposure to sun light and metabolized in the liver to its active form (1α-25-dihydroxy-vitamin D3). Recent evidence suggests that low levels of vitamin D3 are associated with the onset of NAFLD and its severity, independently of other confounding factors. Although the molecular mechanism underlying this association is not yet fully known, it has been shown that the active form of vitamin D (1α-25-dihydroxy-vitamin D3) modulates the pathway of the response to insulin, suppress the proliferation of fibroblasts and the production of collagen and inflammatory pathways.

**References**

1) Neuschwander-Tetri BA, Calwell SH. Nonalcoholic steatohepatitis: summary of an AASLD Single Topic Conference. Hepatology 2003;37:1202-1219.

2) Nobili V, Comparcola D, Sartorelli MR, Natali G, Monti L, Falappa P, Marcellini M. Blind and ultrasound-guided percutaneous liver biopsy in children. Pediatric Radiology 2003;33:772-775.

3) Kleiner DE, Brunt EM, Van Natta M, Behling C, Contos MJ, Cummings OW, Ferrell LD, Liu YC, Torbenson MS, Unalp-Arida A, Yeh M, McCullough AJ, Sanyal AJ. Nonalcoholic Steatohepatitis Clinical Research Network. Design and validation of a histological scoring system for nonalcoholic fatty liver disease. Hepatology 2005;41:1313-1321

4) Nobili V, Pastore A, Gaeta LM, Tozzi G, Comparcola D, Sartorelli MR, Marcellini M, Bertini E, Piemonte F. Glutathione metabolism and antioxidant enzymes in patients affected by nonalcoholic steatohepatitis. Aliment Pharmacol Ther. 2006 Dec;24(11-12):1553-61.

5) Targher G, Scorletti E, Mantovani A, Byrne CD. Nonalcoholic Fatty Liver Disease and Reduced Serum Vitamin D 3 levels. Metab Syndr Relat Disord. 2013 Jun 7. [Epub ahead of print]

6) Roth CL, Elfers C, Kratz M, Hoofnagle AN. Vitamin d deficiency in obese children and its relationship to insulin resistance and adipokines. J Obes. 2011;2011:495101

7) Wortsman J, Matsuoka LY, Chen TC, Lu Z, Holick MF. Decreased bioavailability of vitamin D in obesity. Am J Clin Nutr. 2000 Sep;72(3):690-3. Erratum in: Am J Clin Nutr. 2003 May;77(5):1342.

8) Nobili V, Alisi A, Della Corte C, Risé P, Galli C, Agostoni C, Bedogni G. Docosahexaenoic acid for the treatment of fatty liver: Randomised controlled trial in children. Nutr Metab Cardiovasc Dis. 2012 Dec 7. doi:pii: S0939-4753(12)00256-6. 10.1016/j.numecd.2012.10.010.

**AIM OF THE RESEARCH**

The evidence available thus provide the rationale for the use of DHA and Vitamin D in children suffering from non-alcoholic steatohepatitis, the aim of reducing the oxidative stress in load of hepatocytes and slow the progression of the fibrotic process to load of the liver parenchyma. It is therefore proposed to recruit patients aged between 4 and 16 years, with non-alcoholic steatohepatitis (NASH, documented histologically), with good liver function, to a randomized double-blind study providing for the administration of a mixture containing DHA and vitamin D vs. a placebo for a period of about 6 months (24 weeks).

**PLAN OF RESEARCH AND DETAILED DESCRIPTION OF THE OBJECTIVES AND OF CLINICAL GENERAL AIM:**

**Study Design:** This is a randomized, prospective single-center study, versus placebo in double-blind that can be classified as a Phase III study.

**Research objectives**

Efficacy and tolerability of treatment with DHA and vitamin D in children with nonalcoholic steatohepatitis:

- Evaluating the effectiveness of DHA + Vitamin D in improving 'NAFLD Activity Score' (NAS) of the disease

- Evaluation of the tolerability of the administration of DHA and vitamin D

- Evaluating the effectiveness of DHA and vitamin D in improving haematochemical values 'marker' of the evolution of the metabolic syndrome

**Primary endpoint:** the reduction of the score of the disease in the experimental group compared to the control group.

**POPULATION STUDY:**

**total population:** 66 (33 per randomization arm)

**Their characteristics:** paediatric patients suffering from non-alcoholic steatohepatitis

**healthy volunteers:** no

**Hospitalized patients:** no (admitted to outpatient care)

**outpatients:** yes

**Minor:** yes

**Gender:** Both

**Age:** 4-16 years

**THE STUDY AND 'POLYCENTRIC?** NO

**Inclusion Criteria:**

Biopsy consistent with the diagnosis of NAFLD/NASH

Reduced serum levels of vitamin D aminotransferases (ALT) levels <10 upper limit of normal

hyperechogenicity at liver ultrasound examination suggestive of fatty liver

International normalized ratio (INR) < 1,3 Albumin > 3 g/dl total bilirubin < 2,5 mg/dl

No previous gastrointestinal bleeding

No previous portosystemic encephalopathy

Normal renal function

No hepatitis B, hepatitis C infection

Normal cell blood count

**Exclusion Criteria:**

Alcohol consumption

Use of drugs known to induce steatosis or to affect body weight and carbohydrate metabolism

Autoimmune liver disease, metabolic liver disease, Wilson's disease, and a-1-antitrypsin-associated liver disease

Every clinical or psychiatric disease interfering with experimentation according to investigator's evaluation

Finding of active liver disease due to other causes

**CRITERIA FOR THE INTERRUPTION OF TREATMENT:**

Adverse events

All adverse events which occurred during the course of the clinical study will be reported in the case report form that will be completed for each individual patient. An adverse event is defined as any adverse change with respect to the patient's pre-treatment basal conditions regardless of whether the event is or is not considered treatment-related. This includes all relevant laboratory abnormalities and new illnesses that occur or worsen during the treatment period. It is considered treating any medication administered during the study. The intensity of adverse events will be evaluated using a 3-point scale (mild, moderate, severe). It must also be always documented the relationship between the adverse event and the treatment in progress.

| Severity of adverse events | |
| --- | --- |
|  | |
| Mild | You notice a malaise, but do not have any impairment of normal daily activities |
| Moderate | Malaise enough to reduce or affect normal daily activities |
| Severe | Incapacitating with inability to work or perform normal daily activities |
| Life-threatening | It represents an immediate threat to the patient's life |

Any adverse reactions will also be classified according to the "grading" NCI / NIH (see Annex). In case of serious adverse events, the Ethics Committee will be warned.

Laboratory tests on the safety of the treatment:

- The toxicity data be evaluated based on the exams required by the study design.

The treatment will be suspended the following conditions:

- Toxicity of grade III-IV according to the NCI / NIH criteria;

- If the patient expressing the intention to stop treatment

The expected period of the Experiment: 12 months in total including six months for enrollment and 24 weeks in patient enrolled.

SCHEDULED DATE FOR START OF EXPERIMENTS: October 2013

**TYPE OF STUDY AND EXPERIMENTAL DESIGN AND ITS DESCRIPTION:**

Study: prospective, randomized, Phase III, placebo, with double blindness.

The patients will be randomized into two groups to receive oral emulsion of docosahexaenoic acid (DHA) + Vitamin D3 for oral use pearls (the experimental group), or placebo (control group) that will be made of an inert substance ( corn oil) of that aspect of the pearls of DHA and vitamin D, and will be provided with the product KRILLING D from Italchimici. The requirements concerning the arrangements for daily administration and conservation of the drug will be provided by the Unit of Hepatometabolic Diseases of our Hospital to parents at enrolment.

The assessment of candidates to the inclusion in the protocol in question will be carried out by the Medical of Unit of Hepatometabolic Diseases.

The formulation of the suitability will be based on clinical assessment and the instrumental and laboratory investigations performed. At enrolment in the protocol, we will ask the parents to sign the attached consent form informed the study.

The expected duration of treatment is 24 weeks (approximately 6 months). All patients (and those randomized to the pearl of DHA and vitamin D, both those in the control arm) will be followed every 6 months at our Hepatometabolic Diseases and will undergo check-up, serological and instrumental exams. At the time of study and each semester it will be collected a blood sample for serum bank.

If any patient’s toxicity will not be logged continue the treatment until the end of the period. In case of onset of toxicity or some serious adverse event in some patients, the study will be immediately suspended. Should patients experience an increase in transaminases (equal to or> 6 x N), will be subjected to a clinical and laboratory control with monthly dosing of liver function tests (GOT, GPT, total and fractionated bilirubin, alkaline phosphatase, gamma GT, proteinaemia total, albumin, Quick time and immunoelectrophoresis protein).

**PRODUCT:**

Number of investigational products: 1

Proposed ICD code: 571.8

proposed indication**:** TREATMENT OF PATIENTS IN ETA 'PEDIATRIC AFFECTED BY NONALCOHOLIC STEATOHEPATITIS.

**PRODUCTS DATA OF EXPERIMENT:**

- Dietary product for special medical purposes: Krilling D®
- Pharmaceutical product: dietary product
- Active ingredient: docosahexaenoic acid + Vitamin D3
- Dosage: 225 mg DHA + 800 IU of Vitamin D
- MAH: Italchimici
- Producer Company: Italchimici
- Pharmaceutical Form: pearls
- Route of administration: Oral
- maximum treatment duration for experimentation: 24 weeks (6 months)
- Any association with drugs with the same indication: No
- Concomitant drug therapies with drugs with other indications? NO
- Comparison type:
- Other medications: NO
- Placebo: YES
- The same drug to clinical assays: NO

**Other (please specify):** All patients will be prescribed a balanced low-calorie diet (25-30 cal / kg / d; carbohydrates 50-60%; 23-30% fat, 15-20% protein, fatty acids: saturated 2/3 and 1/3 unsaturated; ω6 / ω3 ratio = 4: 1).

In addition to all patients it will be recommended an activity 'physical program of 30 minutes a day, at least 3 times a week.

**EVALUATION OF THE TREATMENT:**

The primary efficacy parameter is the normalization of transaminases and reduced liver fibrosis, documented by liver biopsy.

**TOXICITY, ADVERSE REACTIONS ADVERSE EVENTS OR PROBABLE & POSSIBLE SECOND STUDIES ALREADY 'MADE:**

The administration of DHA is generally well tolerated. Supplementation of vitamin D is generally well tolerated when taken at recommended doses. The main adverse effects are in fact linked to an overdose resulting in hypercalcemia and its complications.

**PROCEDURES FOR REPORTING AND REGISTRATION OF ADVERSE EVENTS AND / OR ADVERSE REACTIONS**: Adverse events tab

**METHOD FOR MANAGEMENT, EVALUATION AND PROCESSING AND DISTRIBUTION OF PRELIMINARY AND FINAL DATA:**

At the screening visit (T0), the following controls are provided:

- Complete medical history;

- Complete physical examination;

- Blood count with differential;

- Liver function parameters: AST, ALT, total and fractionated bilirubin, alkaline phosphatase, gamma GT, albumin, total protein and electrophoresis proteinaemia, INR, total cholesterol, triglycerides, blood glucose, alpha-fetoprotein, LDH;

- Renal function parameters: blood urea nitrogen and creatinine;

- Electrolyte balance: Sodium, potassium, calcium and magnesium;

- serum iron and ferritin

- OGTT;

- Vitamin D

- Ultrasound abdomen.

The T1 visit, carried out after 6 months of treatment, will be performed the following investigations:

- Clinical evaluation;

- blood exam completed;

- Liver function parameters: AST, ALT, total and fractionated bilirubin, ALP, gamma GT, albumin, total protein and electrophoresis proteinaemia, INR, total cholesterol, triglycerides, blood glucose, alpha-fetoprotein, LDH;

- Vitamin D values

- 5 ml of blood for serum bank.

At the end of study, performed after 12 months of study, we will be performed the following investigations:

- Clinical evaluation;

- blood exam completed;

- Liver function parameters: AST, ALT, total and fractionated bilirubin, alkaline phosphatase, gamma GT, albumin, total protein and electrophoresis proteinaemia, INR, total cholesterol, triglycerides, blood glucose, alpha-fetoprotein, LDH;

- Vitamin D values

- 5 ml of blood for serum bank

-Liver biopsy.

**DATA ANALYSIS AND EVALUATION OF RESULTS:**

Sample Size and Statistical Analysis

Statistical tests: Fisher's exact test

Type I error (Alpha) 0.05

Probability (Pi): 0.10 vs 12:45

sample size (n): 33 per arm

Power = 83.00%

***

Consider that 10% of the placebo group reduces the NAS to values ​​<2 (1.2) and that with 30 patients per group will have an 83% power to declare statistically significant at a 0.05 alpha level an absolute difference 35% of the response (45% experimental group - 10% of the placebo group).

Whereas a 10% drop-out, the number of final patients to be enrolled will be equal to 66.

Randomization

Randomization is achieved by the use of random number tables, the sequence is generated by the computer with allocation in a sealed envelope (epidemiology and statistics service). The key to encode the patient will be taken from the pharmacy, as the doctor in charge will remain blinded to the allocation of the patients in the two arms, one with mixture of DHA and vitamin D, and the placebo.

The analysis will be "intention to treat", understood each patient in the original randomization arm.

**ANALYSIS OF RELATIONSHIP BENEFIT / RISK:**

Based on the results published in the literature and the experience of the proposing groups, treatment with DHA and vitamin D appears to be very well tolerated, and are not described significant side effects.

**MEASURES FOR THE PROTECTION OF THE INDIVIDUAL RIGHTS:**

Parents or legal guardians who accept that their child is included in the protocol, will be asked to sign the attached Informed Consent (OPBG module) before the study. The consent form is accompanied by an Information Card for parents or the patient's legal guardians.

The acceptance to participate in the study may be terminated without notice or obligation on the part of the patient who will be made aware of these abilities prior to accession to the protocol.

The collection of data regarding the possible toxicity of medical care will be unaware of the randomization arm belongs to the patient.

**IDENTIFICATION OF THE STRUCTURE IN WHICH THE TRIAL WILL BE 'MADE:**

Unit of Hepatometabolic disease of Bambino Gesù Children’s hospital IRCCS, 00165- Rome

Referral physicians for the study:

Unit of HepatoMetabolic Diseases - Bambino Gesù Children’s Hospital IRCCS in Rome

Chief Dr. V. Nobili

Tel: 06/68592243
